# A fine-tuned yeast surface-display/secretion platform enables the rapid discovery of neutralizing antibodies against *Clostridioides difficile* toxins

**DOI:** 10.1186/s12934-023-02200-4

**Published:** 2023-09-25

**Authors:** Ying Sun, Yongrong Zhang, Hua Yu, Ashley Saint Fleur, Di Yu, Zhiyong Yang, Hanping Feng

**Affiliations:** 1 Department of Microbial Pathogenesis, School of Dentistry, University of Maryland, Baltimore, MD 21201 USA; 2 Department of Pathogen Biology, School of Basic Medical Sciences, China Medical University, Shenyang, 110122 China; 3 Fzata, Inc, Halethorpe, MD 21227 USA

**Keywords:** Antibody secretion, Antibody surface display, Neutralizing antibody, Infectious diseases, Toxins

## Abstract

**Background:**

Neutralizing antibody plays a key role in protecting hosts from invasive pathogens and their virulent components. Current high-throughput assays for antibody screening are based on binding activities. However, those antibodies with high affinity may not have neutralizing activities. Subsequent functionality assays are necessary to identify neutralizing antibodies from binders with high affinity to their target antigens, which is laborious and time-consuming. Therefore, a versatile platform that can rapidly identify antibodies with both high binding affinity and neutralizing activity is desired to curb future pandemics like COVID-19.

**Results:**

In this proof-of-concept study, we adapted *Saccharomyces cerevisiae* to either display human antibodies on the yeast surface or secrete soluble antibodies into the cultivation supernatant under a controllable ‘switch’ through different carbon source induced promoters. Initially, an engineered chimeric-bispecific Fab antibody, derived from humanized nanobodies against both *Clostridioides difficile* toxin A and B (TcdA and TcdB), was successfully expressed either on the yeast cell surface or in the culture medium with intact bioactivity, suggesting the applicability of our system in antibody display and secretion. Next, a combinatorial Fab library was constructed from B cells isolated from a convalescent patient with a high serological neutralizing titer against TcdB. Following three rounds of magnetic bead enrichment and one round of flow cytometry sorting, antibodies against TcdB were enriched efficiently. We then sorted out single binders with high binding affinity and induced them to express soluble antibodies in culture medium. The neutralizing activity of culture supernatant was analyzed using cell-based assay immediately. This way, we rapidly identified two unique neutralizers (out of seven binders) that can neutralize the cytotoxicity of TcdB.

**Conclusion:**

The antibody screening platform described here simplifies the neutralizing antibody discovery procedure and will be an attractive alternative for screening functional antibodies against infectious diseases.

**Supplementary Information:**

The online version contains supplementary material available at 10.1186/s12934-023-02200-4.

## Introduction

Combinatorial antibody libraries displayed on a variety of cell surfaces allow for the isolation of antibodies with high affinity and specificity for almost any targets. The utilities of these display platforms were validated by the identification of many potent antibodies to treat human diseases [1, 2]. The yeast surface display (YSD) platform has been widely used in antibody screening, protein engineering and characterization, and protein epitope mapping etc., since its initial development by Border and Wittrup in 1997 [3–7]. Yeast is a eukaryotic species that has a superior ability over bacteria and phage display platforms to produce proteins from mammalians given its eukaryotic protein folding and secretion machinery [3].

**Fig. 1:**
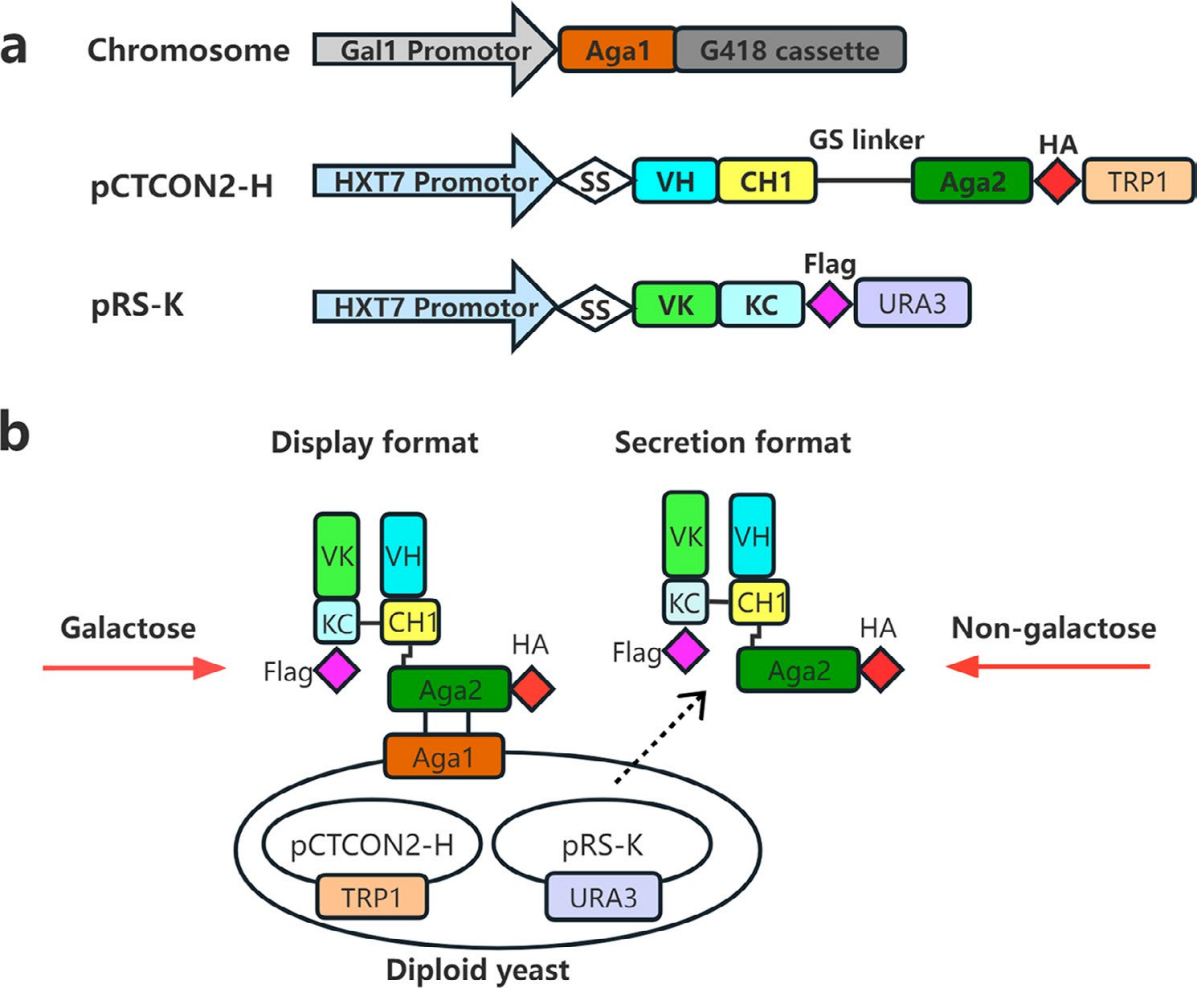
Schematic representation of Fab display or secretion platform. (**a**) The encoding gene cassettes of Aga1, variable region-HC-Aga2 and variable region-LC. The gene encoding Aga1 is located on a chromosome. The genes encoding HC and LC are located on two separate plasmids. (**b**) Fab display and secretion. Diploid yeast harboring both plasmids encoding HC and LC will display Fab on cell wall via Aga1-Aga2 interaction when induced by galactose but will secrete Fab into the culture supernatant when a carbon source other than galactose is present

In the YSD platform, the antibody variants are fused to yeast surface proteins [4, 8]. Aga1 and Aga2, belonging to the a-agglutinin family, are a typical pair of surface anchor proteins in yeast [4, 9]. Aga2 serves as a carrier vehicle and transports the expressed protein of interest (POI) to the anchor protein Aga1 in the yeast cell wall [4]. However, the biggest pitfall of using yeast for antibody screening is the library size limitation due to transformation efficiency [10–13]. In order to increase the antibody diversity and enlarge the library size, fragment antigen-binding (Fab) regions are displayed on the yeast surface [9, 11]. Fab antibody display relies on the generation of plasmids encoding either heavy chain (HC) or light chain (LC) fusion proteins individually in haploid yeast strains. These haploid yeast cells can be mated into diploids, subsequently encoding light chain and Aga2 fused heavy chain in single yeast cells (Fig. 1) [9, 11, 14]. Upon co-expression of both chains, assembly of the heterodimeric heavy and light chain fragment occurs leading to cell-surface display of Fab via Aga1-Aga2 covalent interaction [15, 16].

Therapeutic antibodies have become promising drugs in the past decades due to their low toxicity and high specificity, targeting a wide range of diseases [2]. In recent years, neutralizing antibodies (nAbs) have opened up a new era to targeting infectious diseases especially infections caused by antibiotic resistant bacteria [1]. The nAbs targeting bacterial virulent components, such as *Clostridioides difficile* toxins, and tetanus neurotoxin, or virus surface proteins, such as ebolavirus glycoproteins and spike protein of severe acute respiratory syndrome coronavirus-2 (SARS-CoV-2), have shown superior efficacy in protecting the host from the infections [17–21].

Antibodies with desired protective functionalities are a rare component of an antigen specific antibody library. Since the binding affinity does not correlate to neutralizing activities of the candidate binders, the canonical antibody screening procedure often requires molecular cloning and purification of the selected binders for subsequent functionality assays [22–24]. The neutralizing activity is commonly measured via in vitro bioassays which are sensitive and forthright [25–27]. Antibodies expressed in the crude yeast culture supernatant can be used for those assays, eliminating the need for purification [28]. Therefore, the antibody screening platform that can display and secrete Fab simultaneously will accelerate neutralizing antibody discovery, which is a distinct advantage especially in the pandemic situation. Towards this goal, based on the YSD system, we have developed a versatile antibody screening platform that is capable of tuning the surface display and soluble secretion of human Fab libraries, consequently accelerating the discovery of neutralizing antibodies against infectious diseases.

In the Aga1-Aga2 YSD system, the engineered *S. cerevisiae* strain EBY100 carries the *Aga1* gene cassette in its chromosome and is able to express Aga1 protein under the control of the galactose-inducible GAL1 promoter (P_*GAL1*_) [3]. Our platform has introduced the HXT7 promoter (P_*HXT7*_) into the LC and HC-Aga2 encoding plasmids. P_*HXT7*_ drives expression in the absence or a low level of glucose to achieve the secretion of the POI [29]. In our system, with galactose in the culture medium, both P_*GAL1*_ and P_*HXT7*_ are activated and Fab is displayed on the yeast surface while when sucrose is present as the sole carbon source, P_*GAL1*_ is repressed and Fab-Aga2 is efficiently secreted into the culture medium. Thus, the secretion or display of the intact Fab is achieved by using varied carbon sources. Utilizing this yeast surface-display/secretion (YSDS) system, we have rapidly identified two human antibodies that are able to bind and neutralize the cytotoxicity of *C. difficile* TcdB.

## Materials and methods

### Yeast strains

The yeast strain EBYG418 of mating type a (MATa) with three auxotrophic markers (Leu−, Trp−, and Ura−) was the host strain for the HC library. The strain was derived from EBY100 (MATa AGA1::GAL1-AGA1::URA3 ura3-52 trp1 leu2Δ1 his3Δ200 pep4::HIS3 prb1Δ1.6R can1 GAL (ATCC® MYA-4941) [3]. Compared to EBY100, EBYG418 was engineered to carry the G418 selective marker as previously described [9]. Briefly, the URA3 gene was knocked out via homologous recombination using the gene fragment encoding G418 flanked by 50 bp of homologous gene. The host of the LC library is strain YVH10 (ATCC® MYA­4940TM) of mating type α (MATα) with two auxotrophic markers (Trp − and Ura−).

### Media

EBY100 was cultivated in yeast peptone dextrose broth (YPD). EBYG418 was cultivated in YPD supplemented with G418 (Corning). Yeast haploid cells with HC or LC library and yeast diploid cells with Fab library were cultivated in yeast synthetic minimal media: yeast nitrogen base without amino acids, with yeast synthetic drop-out medium supplements (-URA and/or -TRP) according to requirement, and a carbon source (if supplemented with glucose then “SD” medium). For induction of Fab library, yeast cells were transferred into medium wherein the glucose carbon source was replaced by galactose (SG) for surface display, or sucrose (SS) for secretion. Except where noted chemicals were purchased from Sigma.

### Vectors

To generate the HC expression plasmid pCTCON2-H (Additional file 1: Fig.S1A), shuttle vector pCTCON2 with TRP1 selection marker was modified (pCTCON2 was a gift from Dane Wittrup, Addgene plasmid # 41,843; http://n2t.net/addgene:41843; RRID:Addgene_41843). A gene fragment containing HC constant region 1 (CH1), including the cysteine residue at the last position, fused with Aga2-HA tag at its C-terminus, was synthesized and cloned into pCTCON2 using Gibson Assembly master mix (New England Biolabs (NEB)). *P*_*HXT7*_ was amplified by PCR using an in-house plasmid as template and cloned into pCTCON2-H between KpnI and EcoRI restriction enzyme sites.

For the construction of the LC expression plasmid pRS-K (Additional file 1: Fig.S1B), the CEN-based shuttle vector pRS416 with URA3 selection marker was modified (ATCC® 87521TM). Multiple cloning sites were introduced into pRS-K vector through Gibson Assembly. *P*_*HXT7*_, kappa chain constant region (KC), and cyc1 terminator were amplified respectively and ligated into pRS416 vector via corresponding restriction enzyme sites.

We introduced two SfiI sites before CH1 and KC so that the HC variable region (VH) and kappa chain variable region (VK) could be inserted into the vectors after digestion with SfiI (NEB). The pre-sequence of α-mating factor secretion signal peptide was synthesized as the protein secretion signal peptide.

### Evaluation of the surface display and secretion of Fab bispecific antibody

The single variable domains on heavy chain only antibodies (VHHs) AH3 and E3 against toxin A (TcdA) and toxin B (TcdB) respectively, have shown significant efficacy in preventing *C. difficile* infection (CDI) [21]. To validate the surface display and secretion of Fab antibody in this platform, AH3 and E3 were inserted into the HC and LC vector through SfiI sites, respectively. EBYG418 carrying pCTCON2-AH3-HC-Aga2 plasmid and YVH10 carrying pRS-E3-KC plasmid were mated to generate diploid strain selected in SD media without uracil and tryptophan (URA-TRP-). Briefly, equal numbers of the haploid cells were mixed, and spread onto the regular YPD agar plate (55 cm^2^) (3 × 10^6^ cells/cm^2^) to incubate at 30 °C for 6 h. The cells were then collected and seeded onto an SD plate (URA-TRP-) to generate diploid cells.

To induce surface display of protein, yeast haploid cells containing pCTCON2-H-AH3 and diploid cells containing both plasmids were cultured in SD selective media overnight at 30 °C. Cells were then diluted in fresh media to an OD600 of 0.1 and cultured for several hours. When the OD600 reached 1.0, the cells were transferred to 2×SG selective media (with galactose as the carbon source) and incubated at 20 °C for 2 days. To evaluate surface display by flow cytometry, the cells were incubated with mouse anti-HA (Abcam, ab18181, 1:1000) and goat anti-human KC (Southern Biotech, 2060-01, 1:1000) followed by secondary labeling with donkey anti-mouse Dylight 550 (Invitrogen, SA5-10167, 1:500) and donkey anti-goat Alexa Fluor (AF) 488 (Invitrogen, A-11,055, 1:500). For secretion, yeast haploid cells containing pRS-K-E3 and diploid cells containing both plasmids were induced in 2×SS selective media at 30 °C for 2–3 days. To evaluate secretion, yeast culture supernatants were used in an in vitro neutralization assay (detailed below) or were immunoblotted and detected with HRP conjugated rabbit anti-HA tag antibody (Invitrogen, PA1-29751).

### VH and VK gene pools preparation

B cells were isolated by EasySep™ Human B Cell Isolation Kit (StemCell Technologies) from PBMC samples. Total RNA was extracted using RNeasy plus mini kit (Qiagen). 1 μg of RNA was used for the cDNA synthesis by QuantiTect reverse transcription kit (Qiagen).

Human rearranged VH and VK genes were separately amplified from cDNA using GoTaq Green Master Mix (Promega). The design of VH and VK domain repertoire primers was based on a comprehensive analysis of the alignment of the corresponding V-region alleles from the IMGT database [30]. The primers with gap repair overhang are listed in Additional file 2: Table S1. For VH amplification, nested PCR was performed with 1 μL cDNA using 10 discrete specific sub-family primers annealing to framework 1 region and 4 reverse primers annealing to J domain. For the first step, the primers without overlapping sequences were used to amplify VH under the following conditions: 95 °C for 120 s, 15 cycles of 95 °C for 30s, 60-52.5 °C (-0.5 °C/cycle) for 40s, 72 °C for 40s, 30 cycles of 95 °C for 30s, 67 °C for 40s, 72 °C for 40s and 72 °C for final extension 5 min. For the second step, the purified VH mix and the VH primers with overlapping sequences were used to amplify the tailed VH under the same conditions. For VK amplification, PCR was carried out by 10 cycles of 95 °C for 30s, 57 − 47 °C (-1 °C/cycle) for 40s, 72 °C for 40s, 30 cycles of 95 °C for 30s, 60 °C for 40s, 72 °C for 40s and 72 °C for final extension 5 min. The density of PCR products was determined by electrophoretic bands analysis using Genetools software [31, 32]. The PCR products of VH, tailed VH and VK were mixed based on statistical analysis of sub-family genes abundance in the IMGT database and then were purified via Qiaquick gel extraction kit (Qiagen).

### Yeast mating

The heavy chain plasmid pCTCON2-H and kappa chain plasmid pRS-K were linearized by restriction enzyme SfiI. The linearized plasmids were mixed with tailed VH or VK amplicon at 1:3 molar ratio and precipitated by Pellet Paint Co-precipitate (Novagen). The precipitated mixtures were resuspended and transformed into EBYG418 or YVH10 by electroporation using a Bio-Rad Gene Pulser electroporation apparatus as previously described [13]. The transformants were cultured in liquid SD selective media based on the carrying plasmid. The VH or VK haploid library size and electroporation efficiency were calculated by plating serial dilutions onto the solid selective medium. Five parallel transformation reactions were implemented for each library construction. Yeast cells treated by Zymolyase (Zymo Research) were used as template for colony PCR to sequence VH and VK genes. To validate surface display of HC antibody library, 94 randomly selected yeast haploid cells were induced in 2×SG (TRP-) and labeled with anti-HA tag antibody, followed by donkey anti-mouse Dylight 550. To validate the LC antibody library, 88 randomly selected yeast haploid cells were cultured in 2 × SD (URA-). The expression of LC in supernatant was detected by ELISA in 96 well plates pre-coated with goat anti-human KC antibody. The detection antibody was HRP conjugated anti-FLAG tag antibody (Sigma, A8592, 1:10000).

Yeast mating was performed as previously described [9, 11]. In our case, 2.5 × 10^8^ haploid cells of each library were spread onto a 155 cm^2^ YPD agar plate (3 × 10^6^/cm^2^ cell density). Yeast colonies were washed off the YPD plate, serial diluted and plated out onto single selective (URA- or TRP-) and double selective agar plates for estimating mating efficiency and library size [11]. To induce Fab antibody secretion, 96 randomly selected yeast diploid cells were cultured in 2×SS media (URA-TRP-). The expression of Fab in the supernatant was detected by ELISA in 96 well plates pre-coated with goat anti-human KC antibody. The detection antibody was HRP conjugated rabbit anti-HA tag antibody.

### Library screening by magnetic-activated cell sorting (MACS) and fluorescence- activated single cell sorting (FACS)

The full-length mutated TcdB (aTcdB) was purified as described previously [33] and biotinylated by EZ-Link NHS-PEG4-Biotin (Thermo Scientific). All samples for MACS were kept at 4 °C or on ice and ice-cold buffer was used as Wittrup et al. described [34]. 10^10^ yeast cells from the freshly revived library were induced in 1 L selective SG media (URA-Trp-) at 20 °C for at least 36 h. 10^10^ induced cells were collected and resuspended in 10 ml PBS buffer containing 2% FBS. Yeast cells were first incubated with 250 μl bare beads (7–10 × 10^9^ beads/mL) (Dynabeads™ MyOne™ Streptavidin T1, Invitrogen) for 30 min as negative selection to remove non-specific binding. Unbound yeast cells were then washed and resuspended in 10 ml PBS containing 2% FBS for positive selection. Biotinylated antigen (bio-TcdB) was added to a final concentration of 100 nM (27 μg /ml) and incubated with the cells on a rotator for 30 min. Yeast cells were washed and resuspended, then incubated with 1 ml beads for 30 min. A magnet (EasySep™ Magnet, StemCell Technologies) was then applied to select out the bead-bound cells. A small sample of the collected cells were serially diluted and plated onto SD (URA-, TRP-) agar plates to estimate the cell number. The collected cells were cultured in 100 mL of selective media with 1X penicillin-streptomycin (Cytiva) overnight at 30 °C. These steps were repeated 3 times to enrich for yeast expressing toxin-specific Fab.

5 × 10^7^ cells of the yeast pools after each round of MACS were induced in SG (URA-TRP-) medium at 20 °C for 36 to 48 h. Then, 10^6^ yeast cells of each pool were labeled by 100 nM bio-TcdB and goat anti-human kappa antibody (1:1000 diluted in PBS) followed by streptavidin-AF488 (Invitrogen, S32354, 1:1000 diluted in PBS) and donkey anti goat-PE (Invitrogen, 31,860, 1:200 diluted in PBS). Finally, yeast cells were washed with PBS and analyzed on an SH800 cell sorter (Sony) to monitor enrichment. After the 3rd round of MACS, 10^4^ yeast cells were sorted from the double positive gating quadrant of yeast cells. Then, these cells were plated onto double selective agar plates to form the single colonies.

### In vitro neutralizing assays

To determine the neutralization activity, yeast supernatants were collected and diluted 4 times in cell culture medium, then mixed with 10 pg/ml TcdB or 50 ng/ml TcdA before applying to Vero cell monolayers in a 96-well plate. Cell rounding was observed by phase-contrast microscopy after 24 h of incubation. A tetraspecific antibody ABAB with neutralizing activity against both TcdA and TcdB was used as a positive control [28].

### ELISA and immunostaining

The expression of Fab in the supernatant of the sorted single clones was detected by ELISA in 96 well plates pre-coated with 0.5 μg/ml TcdB. The detection antibody was HRP conjugated rabbit anti-HA tag antibody. After binding or neutralizing activity was observed, surface expression of the positive colonies was induced in SG (URA-TRP-) medium at 20 °C for 48 h. Yeast cells were stained by 1 μg/ml bio-TcdB followed by streptavidin-AF488. Then, the immunofluorescence was read by Cytation 3 (BioTek).

### Characterization of functional clones in an IgG format

Colony PCR was performed to extract the VH and VK genes of each positive clone. The DNA fragment of VH was amplified by PCR with primers terminated with an upstream AgeI site and a downstream SalI site, and subcloned into AgeI/ SalI digested p19eH-IgG1 vector. DNA fragment of VK was amplified by PCR with primers terminated with an upstream AgeI site and a downstream BsiWI site, and subcloned into AgeI/ BsiWI digested pKc-XN vector. The plasmids were co-transfected into Expi293 cells to transiently express IgG antibody according to the manufacture’s protocol (Gibco, A14635). The IgG antibodies were purified using protein A sepharose (Sigma-Aldrich, GE17-5138-03) for in vitro neutralization activity assays.

## Results

### Construction of HC and LC vectors

Compared to the classic, bi-plasmid, Fab YSD system [9], the most significant modification of our system is the replacement of the original promotors in vectors pCTCON2 and pRS416 with P_*HXT7*_ using Gibson Assembly (Fig. 1 & Additional file 1: Fig. S1). The VH-HC was fused to the N-terminal of Aga2 followed by a HA-tag in the pCTCON2 vector while VK-LC was followed by a FLAG-tag in the pRS vector (Fig. 1). Both plasmids are retained in yeast cells under auxotrophic selective pressure. The genes encoding humanized VHHs AH3 and E3 that are able to neutralize the toxicity of *C. difficile* TcdA and TcdB [21, 35] were used as testing genes and ligated into the pCTCON2 and pRS416 vectors through SfiI restriction sites respectively as described in the method.

### Validation of the platform by inducing chimeric Fab-AH3/E3 expression in a diploid strain

The plasmids pCTCON2-AH3-HC-Aga2 and pRS-E3-KC were transformed into yeast strain EBYG418 and YVH10 respectively followed by yeast mating to generate a diploid strain Y-AH3/E3 harboring both plasmids. To validate whether our platform is suitable to regulate display and secretion of functional Fab antibody, both surface display and soluble expression were induced under different culture conditions as illustrated in Fig. 1b.

**Fig. 2:**
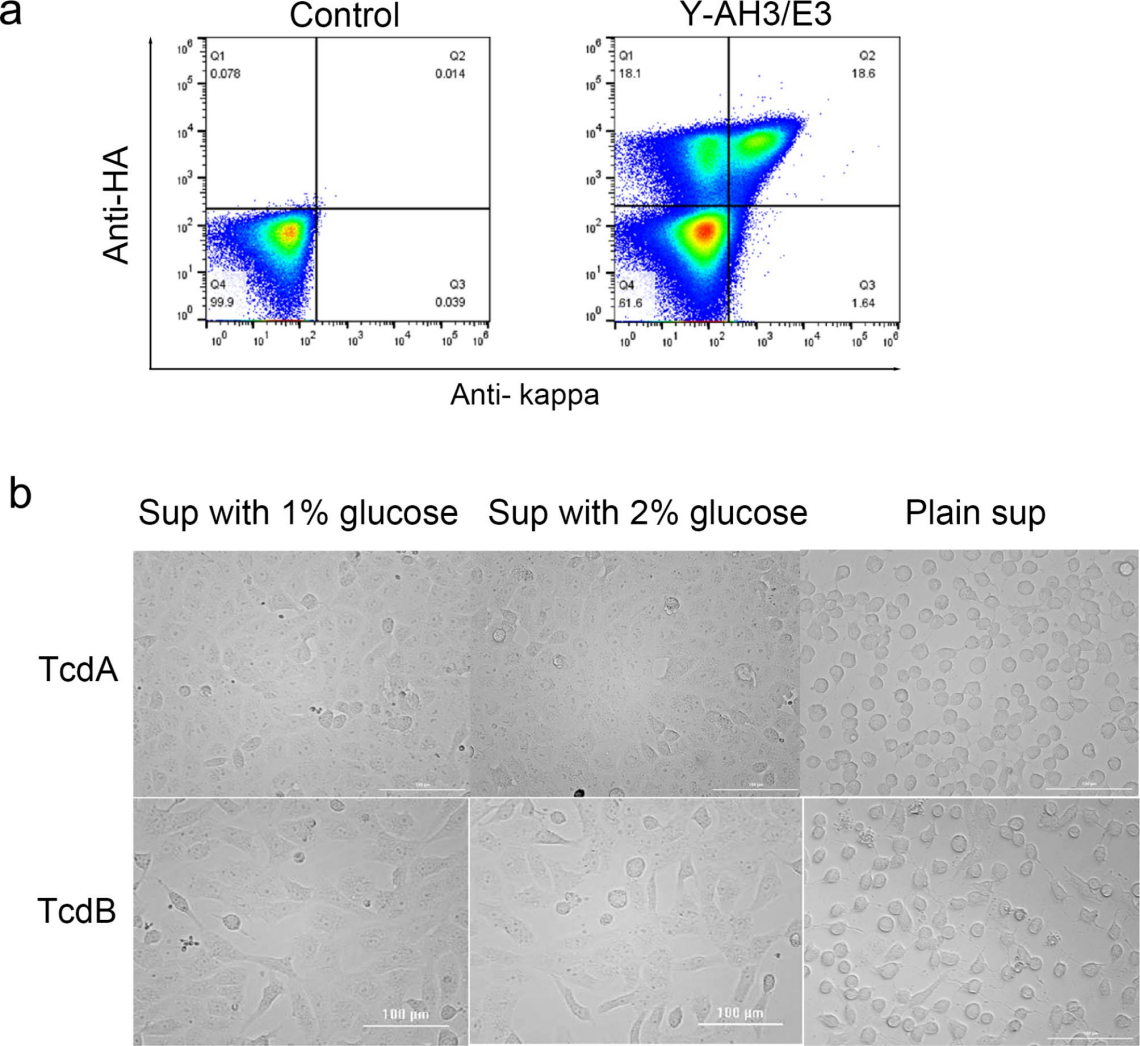
Surface display and secretion of Fab-AH3/E3 in diploid Y-AH3/E3. **a**. The galactose induced yeast cells were harvested and stained to detect Fab display by FACS. The cells were incubated with mouse anti-HA and goat anti-human kappa followed by secondary labeling with donkey anti-mouse Dylight 550 and donkey anti-goat AF 488. The non-induced cells were used as a negative control. **b.** Neutralization assay with secretion culture supernatant. The yeast cells were induced to secrete Fab in SD medium containing 1% or 2% glucose at 30 °C for 72 h. The supernatants were diluted 4 times and co-cultured with Vero cells in the presence of 50 ng/mL TcdA or 10 pg/mL TcdB at 37 °C overnight. The images were taken using a phase-contrast microscope

The diploid yeast Y-AH3/E3 was cultivated in SG (URA-TRP-) to induce surface display. Since Aga1 was expressed on the surface in the presence of galactose, AH3-HC-Aga2 was also displayed on the surface. E3-KC was detectable on the surface when bound to AH3-HC-Aga2 (Additional file 1: Fig. S1b & S2a). In fact, only if E3-KC appeared on surface, was the intact Fab-AH3/E3 displayed. The frequency of E3-KC on the surface was measured by FACS via kappa chain staining while AH3-HC was measured via HA-tag staining. Double positive yeast cells displaying an intact Fab (containing both heavy and light chains) on their surface were about 18.6% of the cultured population as detected by flow cytometry (Fig. 2a), suggesting a successful surface display of the chimeric antibody. However, it is notable that about 18% of the total population were only positive for HA-tag staining but not for kappa chain staining (Fig. 2a). The culture supernatant showed neutralization activity against both TcdA and TcdB (Additional file 1: Fig. S2b), implying leakage of Fab-AH3/E3-Aga2 under the display culture condition. Nevertheless, regarding the neutralizing activity, the leaked amount was not as significant as the secreted amount under secretion conditions (Additional file 1: Fig. S2b).

For soluble expression, instead of galactose, different concentrations of glucose were supplemented in the culture media. In the presence of glucose, *P*_*GAL1*_ was shut down. Thus, no Aga1 was expressed and dimerized Fab-AH3/E3 was secreted into the culture medium. As shown in Fig S2a, when secretion was induced, no detectable Fab signal was seen on the surface. Since both AH3 and E3 are neutralizing antibodies, the soluble Fab-AH3/E3 should be able to neutralize TcdA and TcdB simultaneously. Therefore, the neutralizing activity of the culture supernatants with soluble expression were measured to verify Fab-AH3/E3 secretion. As shown in Fig. 2b, the supernatants cultured with both 1% and 2% glucose for 72 h were able to protect Vero cells from intoxication caused by TcdA and TcdB, indicating the secretion of intact Fab-AH3/E3 with bioactivity. Based on the neutralizing activity of purified VHHs AH3 and E3 respectively, the estimated expression level of the Fab-AH3/E3 was about 1–5 μg/mL.

### Optimized carbon source and temperature for soluble expression

**Fig. 3:**
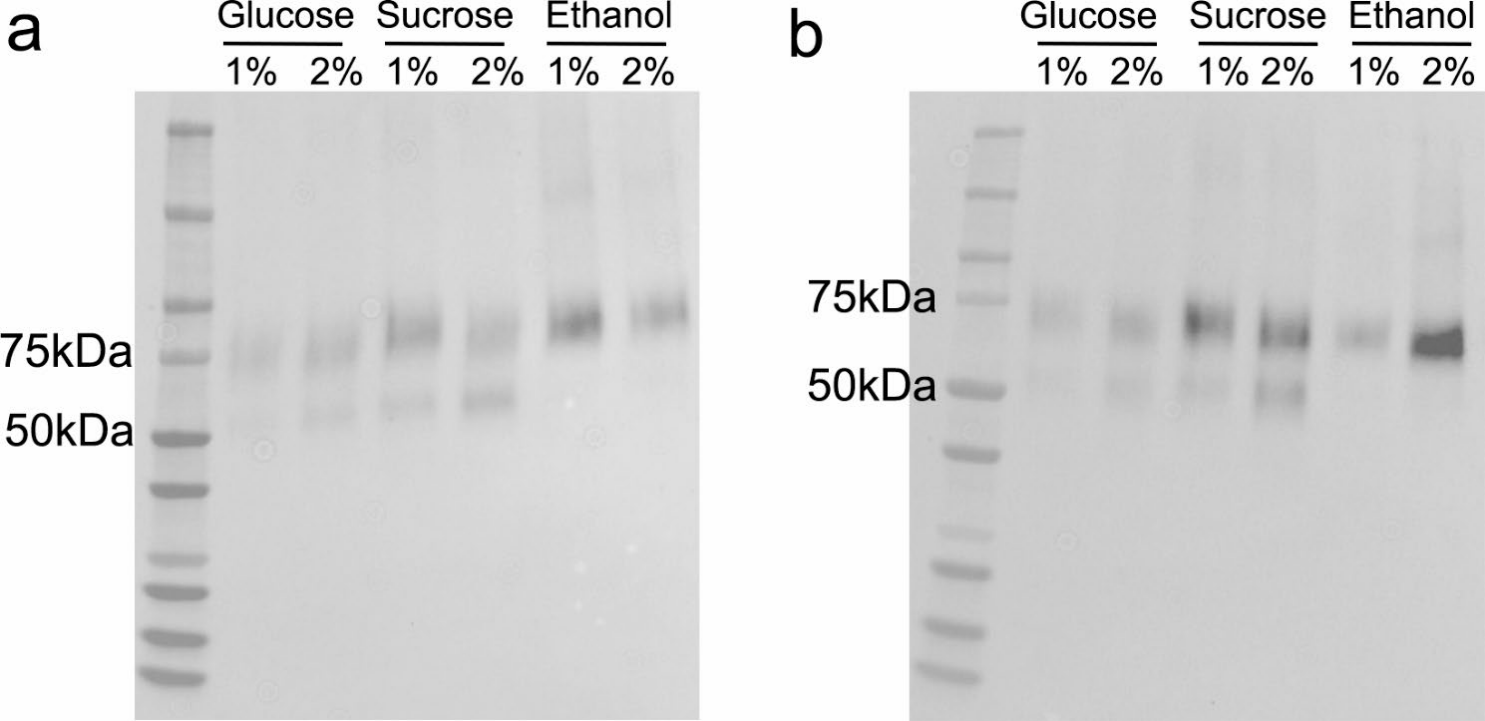
Secretion of Fab-AH3/E3 at different conditions. Y-AH3/E3 cells were induced to produce Fab in synthetic minimal media containing various carbon sources for (**a**) 48 h or (**b**) 72 h as the indicated concentration (w/v). The supernatants were collected, and western blot was performed to detect Fab with anti-HA tag antibody

To achieve an optimal soluble expression level, we modified the culture conditions including carbon source and growth time. The Y-AH3/E3 yeast strain was cultivated in synthetic minimal medium containing different concentrations of sucrose, glucose, or ethanol at 30 °C for 48 h or 72 h. Immunoblotting analysis of the culture supernatants using an anti-HA tag antibody showed the presence of the Fab protein, with greater expression in the sucrose and ethanol supplemented media at 72 h (Fig. 3). Degradation occurred as a lower band was also detected. This degradation may lead to an overestimation of the expression of full length Fab-AH3/E3 by neutralizing assay (data not shown). The endpoint OD600 between samples cultured within the same period were similar except the ones cultured in ethanol, suggesting that the growth was not undermined by selected sugars (Additional file 2: Table S2 & S3). Meanwhile, the ethanol supernatant showed a less potent neutralizing activity against the toxins although it seemed to have more antibody expressed by western blot (Additional file 1: Fig. S2b). Interestingly, 2% glucose showed a better yield of the antibody than the lower concentration of 1%, indicating that at current culture conditions, glucose at an initial higher concentration of 2% does not suppress P_*HXT7*_ after 48 h. Since ethanol affected the growth, therefore, we applied the cultivation condition of 2% sucrose for 72 h as the optimized soluble expression condition for our following studies.

### CDI patient B cell derived Fab library construction and characterization

**Fig. 4:**
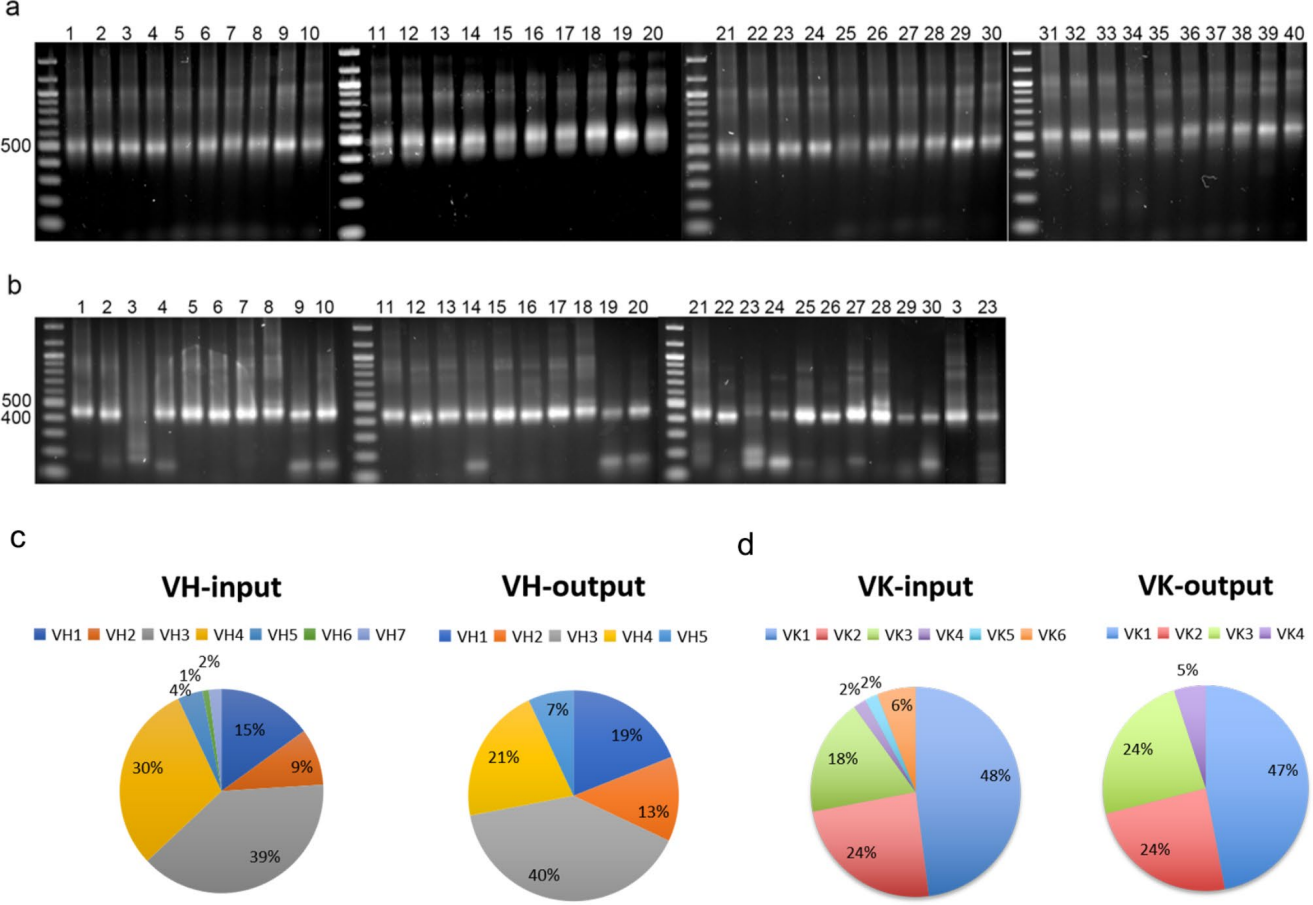
Amplification of VH and VK. 40 PCR products for VH (**a**) and 30 PCR products for VK (**b**) were generated by paired primers. The abundance of each sub-family genes before and after library construction are shown in (**c**)

B cells were isolated from 1.25 × 10^7^ PBMCs of a convalescent patient. cDNA was reverse transcribed from 1 μg mRNA purified from the total RNA extracted from the B cells. A primer set (Additional file 2: Table. S1), designed based on the abundance and frequency of immunoglobulin subfamilies in the IMGT database, was used to amplify VH and VK genes. All the targeted subfamilies of VH and VK were amplified with the optimized PCR conditions (Fig. 4a, b). The corresponding lane numbers of the PCR products from the specific paired primers were listed in Additional file 2: Table. S4 & S5.

The amplified PCR products flanked with homologous arms were then pooled at the indicated ratio in Fig. 4c & d based on sub-family gene abundance and co-transformed with the corresponding linearized vectors into yeast haploid strains. Following five parallel transformations for each haploid library, the calculated library size of VH was about 4.2 × 10^6^ while that of VK was about 2.6 × 10^7^. To evaluate the quality of VH and VK haploid libraries, 68 single clones from the VH library and 56 single clones from the VK library were randomly selected for sequencing analysis. The results revealed that 95% of the sequences from the VH library and 98% of the sequences from the VK library encoded in-frame variable regions of antibodies. Among those sequences, the calculated abundance of the sub-family genes was similar to the input abundance although VH6 and VH7 were not present in the selected clones (Fig. 4c, d). No repetitive sequences were found in the selected clones, indicating high diversity of the generated libraries.

Yeast mating conditions had been optimized to generate a yeast Fab library. In our case, the maximum mating efficiency was about 92% when 1.5 × 10^9^ cells of each haploid library were spread onto 6 YPD plates (155 cm^2^/ plate) simultaneously (3 × 10^6^ cell/cm^2^). The final size of Fab library was about 1.38 × 10^9^ after mating.

Before mating, the VH expressed on haploid yeast EBYG418 surface showed a positive rate as 86% from 94 randomly selected clones by flow cytometry (data not shown). The VK secreted from haploid yeast YVH10 in culture supernatant showed a positive rate of 73% from 88 randomly selected clones by ELISA (Additional file 1: Fig. S3a). Interestingly, compared to haploid libraries, the secretion rate of the Fab library can reach 86% from 192 randomly selected clones by ELISA (Additional file 1: Fig. S3b).

### Screening for nAbs against *C. difficile* TcdB

**Fig. 5:**
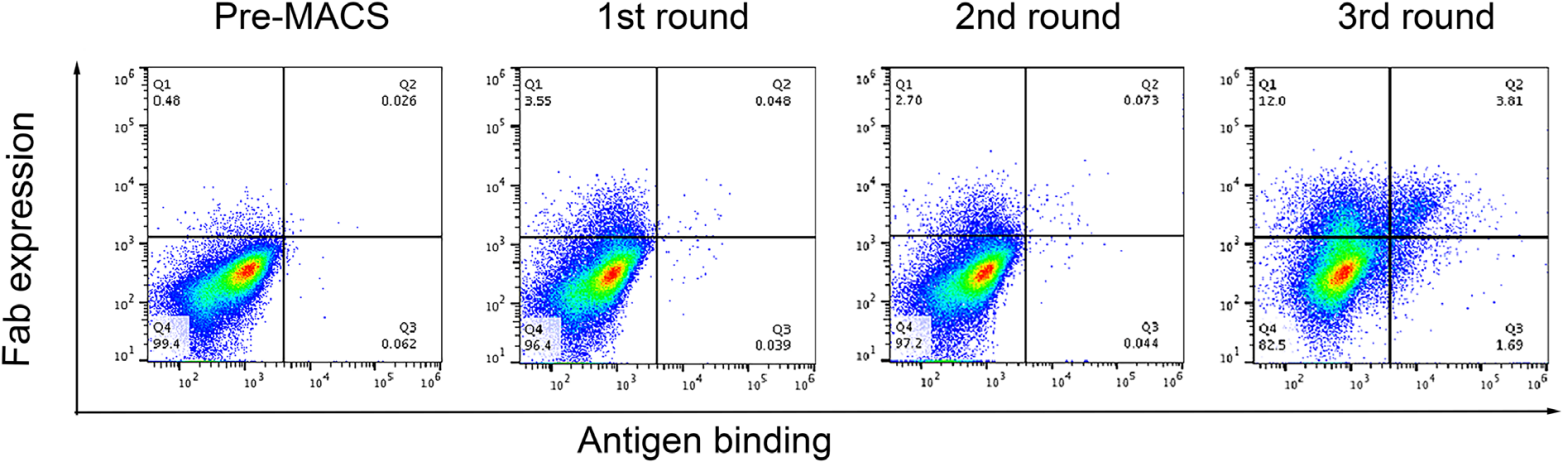
Enrichment of specific binders against TcdB. The library was enriched by MACS for three rounds as described in the methods. After each round, the enrichment was evaluated by FACS. Yeast cells were incubated with 100 nM biotinylated aTcdB (bio-TcdB) and goat anti-human kappa antibody, followed by secondary labeling with streptavidin-AF488 for detecting antigen binding and donkey anti goat-PE for detecting surface display

*C. difficile* TcdB was used as an antigen to screen human nAbs from the library. Firstly, the high affinity binders against TcdB were enriched from the Fab library by 3 rounds of MACS, which is routinely used before FACS to screen antibodies from large YSD libraries [36]. Cells of at least ten-fold the library size were input to enrich for binders. A slight enrichment of those positive for both antigen binding and Fab expression (from 0.048 to 0.073%) were seen by flow cytometry after the first two rounds of MACS (Fig. 5). A significant rise (3.81%, Fig. 5) of double positive events were detected after the third round of MACS although the library size shrunk from 1.34 × 10^9^ to 10^4^, suggesting an enrichment of specific binders and elimination of non-binders.

Next, FACS and cell-based assays were performed to isolate individual binders with high binding affinity and neutralizing activity. For FACS, controls stained with detection antibodies were only used to adjust gates to eliminate nonspecific binding. 4 × 10^4^ cells from the last-round enrichment were input for single cell sorting of individual binders. 376 clones were isolated and induced to secrete the Fabs in culture medium. Among all the clones, 7 of them showed binding affinity to TcdB while only two of them (Clone#3 & #7) showed neutralizing activity against TcdB based on the cell-rounding assay. The encoding genes of the 2 neutralizers were amplified and sent out for sequencing. Interestingly, the two clones exhibited similar VH sequences, and different VK sequences (Additional file 2: Table. S6).

**Fig. 6:**
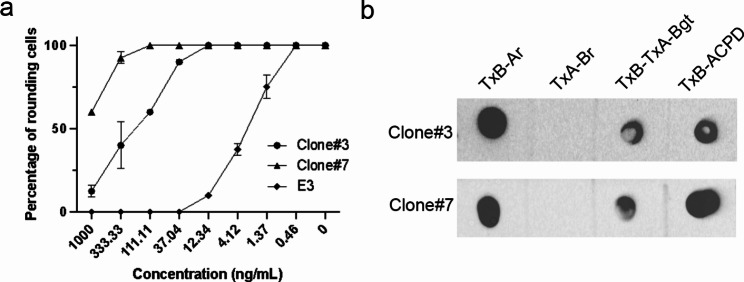
Neutralizing activity and epitope mapping of the identified neutralizers in an IgG format. (**a**) Neutralizing activity assay. Vero cells were co-cultured with the indicated concentrations of the purified IgGs of Clone#3 and #7 in the presence of 10 pg/mL TcdB for 24 h. E3 was included as a positive control. The percentage of rounded cells was calculated under a phase contrast microscope. (**b**) Epitope mapping. Dot blot was performed to identify the epitopes. 200 ng of each chimeric protein was loaded onto the nitrocellulose membrane (Bio-Rad, Cat. RAD-1,620,146). The membranes were then incubated with the purified IgG antibodies of Clone#3 and #7 at a final concentration of 1ug/mL, followed by secondary goat anti-human Fc-HRP (SouthernBiotech, Cat. 2048-05) (1:2000). The image was taken by G-Box Chemi system (Synoptics Ltd, Cambridge, UK)

Afterwards, the two clones were reconstructed into two plasmids for expression of full-length IgG molecules in Expi293 cells. Neutralizing activity of the IgGs was tested by cell rounding assay. As shown in Fig. 6a, the EC50s of Clone#3 was between 111 and 333 ng/mL while that of Clone#7 was about 1000 ng/mL. Four chimeric toxins including TxB-Ar, TxA-Br, TxA-Bgt and TxB-ACPD were utilized to identify the epitopes. Each of the chimeric toxins harbors different domains from TcdB [37, 38]. Both neutralizers were able to recognize TxB-Ar, TxA-Bgt and TxB-ACPD (Fig. 6b). The domain in common of the three chimeric toxins is the glucosyltransferase domain (GTD). Thus, the epitopes of the neutralizers are located in the GTD of TcdB.

## Discussion

Our study described above has proposed a new tunable yeast surface-display/secretion platform for combinatorial antibody library screening which simply relies on supplemental carbon sources to switch the expression format. The classic Aga1-Aga2 YSD fulfilled surface display via the binding of the cell wall ‘anchor’ Aga1 to ‘transporter’ Aga2. The concept of our design was to harness Aga1 expression and thus attain releasing or tethering of Aga2 fused POI. Since the transcription of chromosome integrated *Aga1* gene is driven under *P*_*GAL1*_, therefore, the promoter desired to drive the transcription of the gene of POI is required to be a non-galactose dependent promoter but active in galactose. Besides, similarly to *P*_*GAL1*_, when inducing secretion, a glucose repression promoter is preferred to avoid counterselection against sequences that negatively influence host growth [3]. *P*_*HXT7*_, located in the gene encoding a hexose transporter [39, 40], has a low POI production at the early growth phase when cultivated in medium containing ample glucose [41]. Consistent with Partow et al.’s study, in our study, the HC and LC were not detectable in glucose until culture for 48 h (data not shown). Thus, *P*_*HXT7*_ satisfies all our requirements.

Although the activity of *P*_*HXT7*_ is low in the glucose consuming phase, it escalates significantly when glucose is exhausted and ethanol is being consumed [41]. Therefore, to reach an efficient POI expression, an optimized carbon source other than glucose ought to be pursued. In an earlier study, Peng et al. have demonstrated the efficiency of *P*_*HXT7*_ in a range of industrial carbon sources including glucose, galactose, sucrose, and ethanol. The *P*_*HXT7*_ activity was up-regulated in ethanol, but the POI level was lower than in sucrose and galactose [29]. In our study, although a stronger intensity of POI was detected by western blot, the neutralizing activity of supernatant from ethanol supplemented cultures was not comparable. Besides, ethanol undermined the growth of yeast. By contrast, in sucrose, the secretion level was comparable without affecting yeast growth rate or neutralizing activity. Meanwhile the activity of *P*_*GAL1*_ in sucrose was well suppressed since the surface display could not be detected in yeast growing in sucrose. Interestingly, when inducing surface display using galactose, the neutralizing activity against both toxins was detected in the culture supernatant. The leakage of the antibody under the display condition is possibly ascribed to the excess secretion of Fab over the displayed Aga1. However, the leaked amount of Fab was significantly less than that expressed under the sucrose secretion condition. Since the concentration of the antibody is key to the success of neutralization, thus, independent secretion is still necessary for the screening.

The incentive for proposing this YSDS platform was to accelerate the nAb discovery. Although the phage display platform allows assessing functional activities through supernatants, the misfolding and low secretion efficiency of POI were the major drawbacks compared to the yeast system [1]. Utilizing this YSDS platform, we are able to use the classic YSD to sort out high-affinity binders from a human antibody repertoire and subsequently identify the neutralizers out through a quick functionality assay. Based on our previous experience and others’ [42, 43], the binding affinity of antibodies does not predict their neutralizing activity. In our case, only 2 out of 7 binders showed neutralizing activity against TcdB. Two possible reasons explain the low output of neutralizers. First, the neutralizing titer against the toxins is relatively low among CDI patients although the antitoxin titer is high [43, 44]. The neutralizing titer of the patient in our study was above 300. By contrast, in our previous study, the neutralizing titer of sera from vaccinated animals was able to reach 4*10^3^ [33]. Second, display efficiency will also limit the discovery of nAbs since neutralizers as binders were primarily enriched and sorted out through surface display. After three rounds of MACS, the enrichment rate was relatively low which may also be attributed to the low display efficiency. Co-expression the Fab with protein disulfide isomerase [45], molecular chaperone [46], or improved secretion signal [47], etc. may help to improve the display efficiency of our system in the future.

Previously, two other groups have also proposed YSDS systems for library screening. Unlike ours, the two groups utilized genetic elements to govern the Aga2 fusion, subsequently controlling the display and secretion in yeast. Similar to the phage display system, Deventer and coworkers utilized the amber stop codon and supplementation with a noncanonical amino acid in culture medium to switch Aga2 fusion ‘on’ or ‘off’ [48]. In Cruz-Teran’s system, a self-cleaving 2 A peptide of 50% efficiency was adapted to mediate ribosomal skipping which would produce POI fused with or without Aga2 simultaneously from a single open reading frame [49]. Compared to these two systems, ours has several advantages. The capacity of our platform, referring to the library size, is greater since we adopted a two-plasmid system to encode both heavy and light chains of Fab. As aforementioned, surface display of Fab on diploid yeast is one of the strategies to circumvent technical limitations and enlarge library size. Others also implemented a vector comprising a bidirectional promoter *Gal1/Gal10* to display the Fab antibodies on yeast surface [15, 45]. Although the one-plasmid encoding both heavy and light chain at once will avoid the mating process, this system is not capable of favoring the secretion of the Fab since all target genes were driven under *Gal* promoters. Moreover, our platform is also a potential tool for screening VHH based bispecific antibodies. However, limitations also exist in YSDS platforms for nAb screening. The secretion level of POI of all three platforms was about 1–10 μg/mL which could be the major limitation for some binders needing higher concentrations for neutralization in their bioassays. The display efficiency also needs to be improved since it may determine the diversity of binder pools [45].

## Conclusions

In this study, a yeast surface-display/secretion platform has been developed for rapidly screening nAb against infectious diseases and other targets. The display and secretion of Fab antibody are regulated by varied carbon sources supplemented in the cultivation medium. A high-throughput-based functional assay can be easily achieved in this platform that allows easy and quick identification of functional antibodies directly from yeast antibody libraries. Thus, our study has potentially provided a powerful platform for quicker nAb discovery.

### Electronic supplementary material

Below is the link to the electronic supplementary material.

**Additional file 1: Figure S1**. pCTCON2-AH3 and pRS-K-E3 plasmid maps and schematic representation of Fab-AH3/E3 surface display and secretion. **Figure S2**. Evaluation of Fab-AH3/E3 expression. **Figure S3**. Evaluate the expression of VH, VK and Fab libraries

**Additional file 2: Table S1**. Primers used for VH and VK amplification. **Table S2**. OD600 of Y-AH3/E3 after cultured in various carbon sources for 48 h. **Table S3**. OD600 of Y-AH3/E3 after cultured in various carbon sources for 72 h. **Table S4**. The corresponding numbers of VH PCR products generated by the indicated forward and reverse primers. **Table S5**. The corresponding numbers of VK PCR products generated by the indicated forward and reverse primers. **Table S6**. Gene alignment of the neutralizing clones isolated from the Fab library
