# Supplementary material for: A fine-tuned yeast surface-display/secretion platform enables the rapid discovery of neutralizing antibodies against Clostridioides difficile toxins
Source: Microb Cell Fact. 2023 Sep 25;22:194. doi: 10.1186/s12934-023-02200-4 (PMC10519002; doi:10.1186/s12934-023-02200-4)

**Figure S1. pCTCON2-AH3 and pRS-K-E3 plasmid maps (a) and schematic representation of Fab-AH3/E3 surface display and secretion (b).
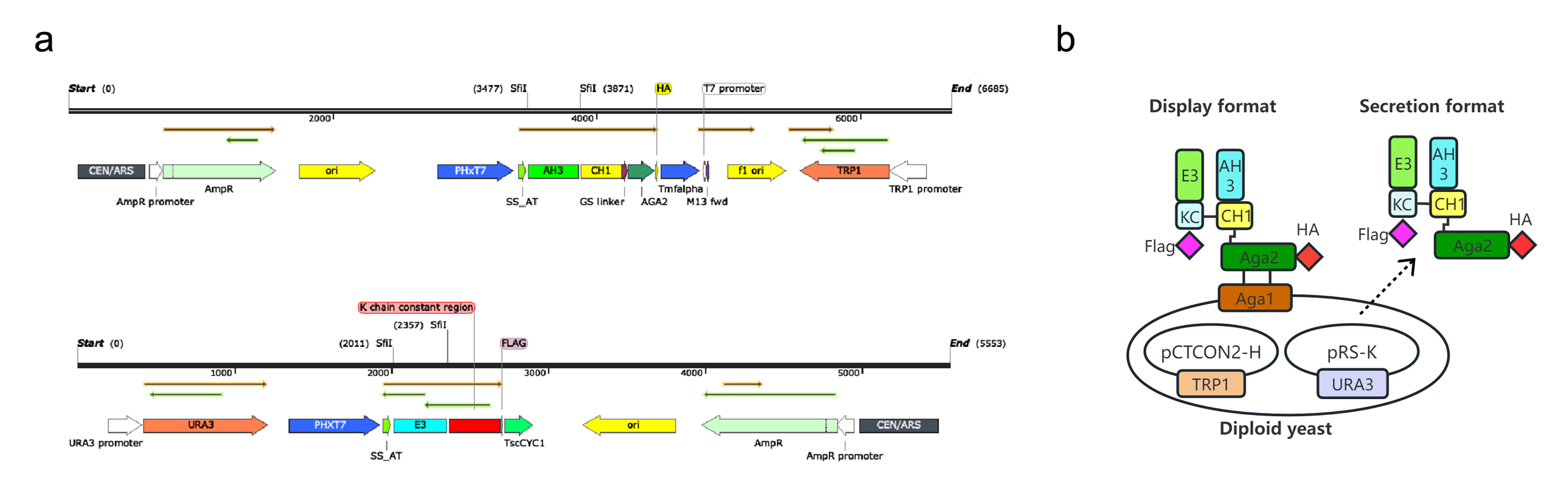
**

**Figure S2. Evaluation of Fab-AH3/E3 expression. (a)** Fluorescent staining of the yeast induced in sucrose (secretion) and galactose (surface display). Yeast cells were incubated with mouse anti-HA and goat anti-human kappa followed by secondary labeling with donkey anti-mouse-Dylight 550 and donkey anti-goat-AF 488. **(b)** Neutralizing activity assay of culture supernatants supplement with 2% glucose, sucrose, ethanol or galactose. The supernatants were serially diluted at 4, 12, 36 and 108-fold and co-incubated with Vero cells in the presence of 50 ng/mL TcdA or 10 pg/mL TcdB for 24h. The percentage of affected cells was calculated under a phase contrast microscope.


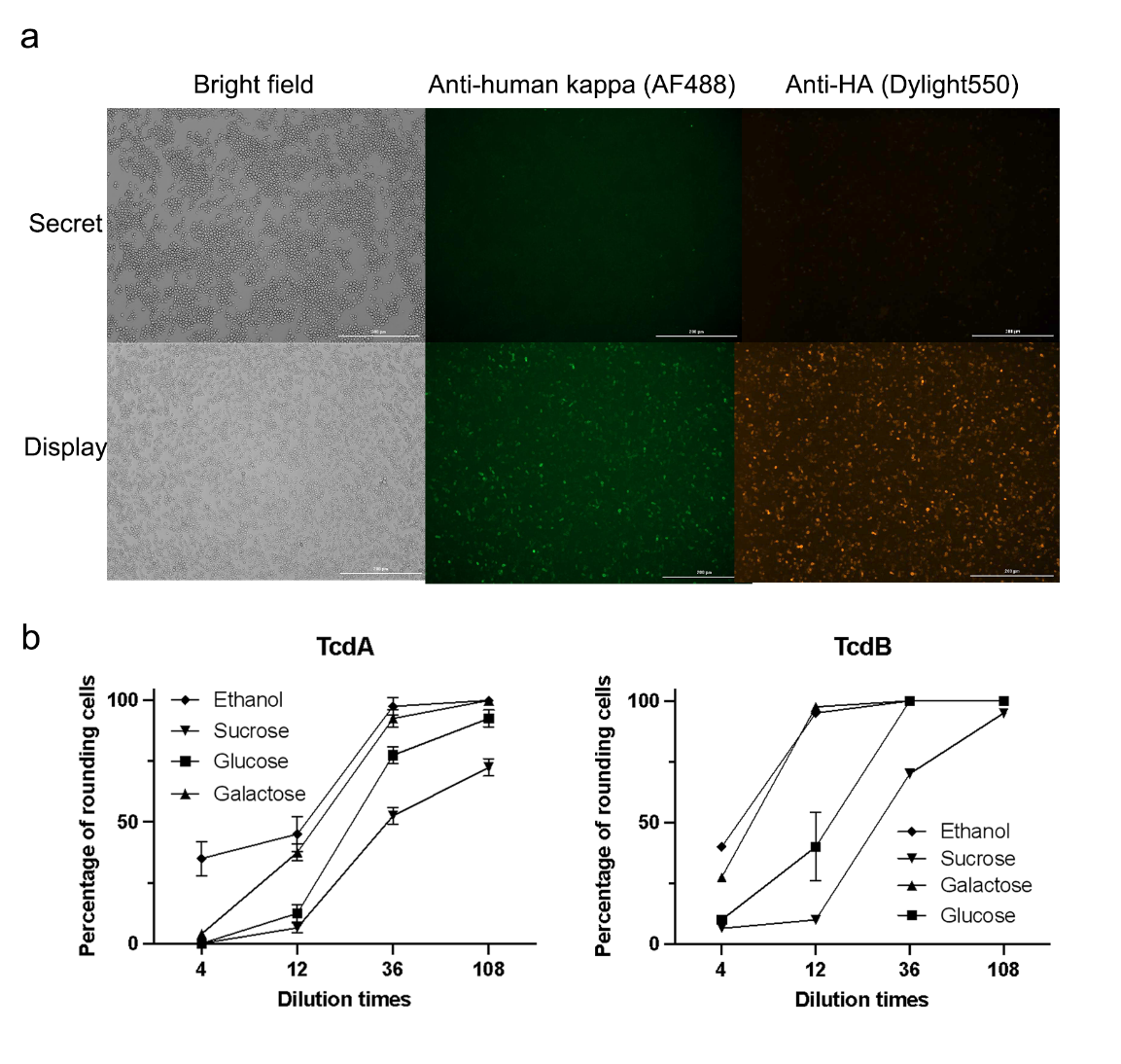


**Figure S3. Evaluate the expression of VH, VK and Fab libraries.** ELISA was performed to measure the secretion of **(a)** VK from 88 haploid clones and **(b)** Fab from 192 diploid clones in the corresponding libraries. The antibodies in the 2-fold diluted supernatant were captured by anti-human kappa antibody and detected by mouse anti-FLAG tag (HRP) for VK library and rabbit anti-HA tag (HRP) for Fab.


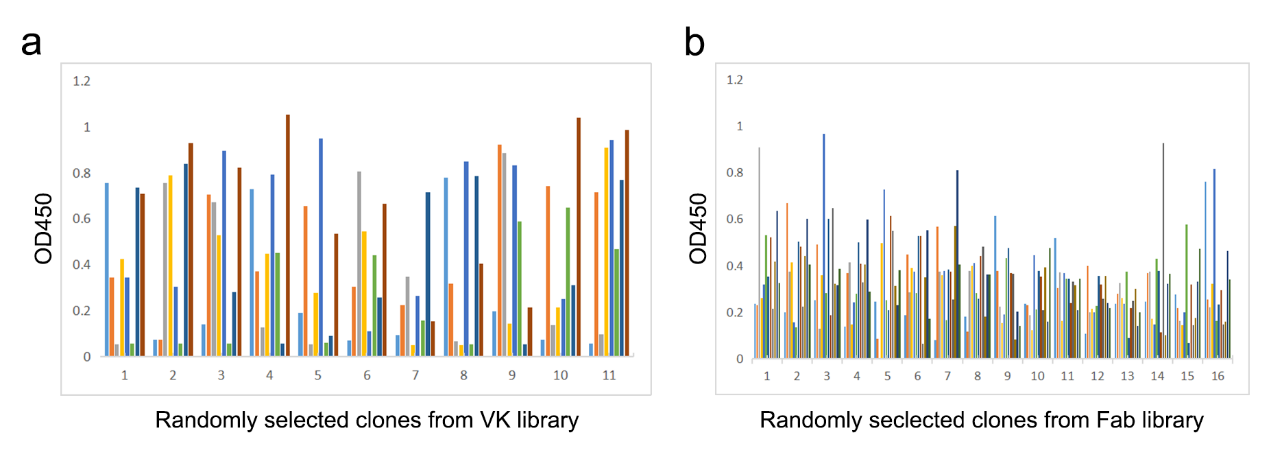

Supplement: Supplementary file 1 — Additional file 1: Figure S1. pCTCON2-AH3 and pRS-K-E3 plasmid maps and schematic representation of Fab-AH3/E3 surface display and secretion. Figure S2. Evaluation of Fab-AH3/E3 expression. Figure S3. Evaluate the expression of VH, VK and Fab libraries [file 12934_2023_2200_MOESM1_ESM.docx]
