# Supplementary material for: A fine-tuned yeast surface-display/secretion platform enables the rapid discovery of neutralizing antibodies against Clostridioides difficile toxins
Source: Microb Cell Fact. 2023 Sep 25;22:194. doi: 10.1186/s12934-023-02200-4 (PMC10519002; doi:10.1186/s12934-023-02200-4)
Supplement: Supplementary file 2 — Additional file 2: Table S1. Primers used for VH and VK amplification. Table S2. OD600 of Y-AH3/E3 after cultured in various carbon sources for 48 h. Table S3. OD600 of Y-AH3/E3 after cultured in various carbon sources for 72 h. Table S4. The corresponding numbers of VH PCR products generated by the indicated forward and reverse primers. Table S5. The corresponding numbers of VK PCR products generated by the indicated forward and reverse primers. Table S6. Gene alignment of the neutralizing clones isolated from the Fab library [file 12934_2023_2200_MOESM2_ESM.docx]

**Table S1**. Primers used for VH and VK amplification

| Primers | | Sub-families covered | Sequences (5’-3’) |
| --- | --- | --- | --- |
| Forward primers  of VH | VH1 | 1/5/7 | CAG RTG CAGCTGGTGCAG |
|  | VH2 | 1 | CAG GTC CAGCTGGTACAG |
|  | VH3 | 2 | CAG RTC ACC TTG AAG GAG TC |
|  | VH4 | 2 | CAG GTC ACC TTG AGG GAG TC |
|  | VH5 | 3 | SAG GTG CAG CTG GTG GAG |
|  | VH6 | 3 | GAG GTG CAG CTG TTG GAG |
|  | VH7 | 3 | GAA GTG CAG CTG GTG GAG |
|  | VH8 | 4 | CAGSTGCAGCTGCAGGAG |
|  | VH9 | 4 | CAGGTGCAGCTACAGCAGTG |
|  | VH10 | 6 | CAGGTACAGCTGCAGCAG |
| Reverse primers  of VH | JH1 | 1/4/5 | TGA GGA GAC GGT GAC CAG |
|  | JH2 | 2 | TGA GGA GAC AGT GAC CAG G |
|  | JH3 | 3 | TGA AGA GAC GGT GAC CAT TGT |
|  | JH4 | 6 | TGA GGA GAC GGT GAC CGT |
| Forward  primers  of VK | VK1 | 1 | GACATCCAGWTGACCCAGTC |
|  | VK2 | 1 | GTCATCTGGATGACCCAGTC |
|  | VK3 | 2 | GATATTGTGATGACCCAGACT |
|  | VK4 | 2 | GATRTTGTGATGACTCAGTCT |
|  | VK5 | 3 | GAAATTGTGTTGACRCAGTC |
|  | VK6 | 3 | GAAATAGTGATGACGCAGTC |
|  | VK7 | 3 | GAA ATTGTG TTGACGCAGTCT |
|  | VK8 | 4 | GACATCGTGATGACCCAGTC |
|  | VK9 | 5 | GAAACGACACTCACGCAGTC |
|  | VK10 | 6 | GAAATTGTGCTGACTCAGTC |
| Reverse primers  of VK | JK1 | 1/2/4 | TTTGAT CTCCASCTTGGTCC |
|  | JK2 | 3 | TTTGAT ATCCACTTTGGTCC |
|  | JK3 | 5 | TTTAAT CTCCAGTCGTGTCC |
| Flanking ^a^ | VH |  | GCTGTTTTATTCGCAGCATCCTCCGCATTAGCTGC |
|  | JH |  | TAAGGGGAATACGCTTGGTCCCTTGGTCGACGCTT |
|  | VK |  | GCTGTTTTATTCGCAGCATCCTCCGCATTAGCTGC |
|  | JK |  | GAAGATGAAGACAGATGGTGCAGCCACCGTACGTT |
| SfiI |  |  | GGCCGGCCTGGCC |

^a^ All primers are flanked with homologous sequences followed by SfiI enzyme site.

|  | Glucose | Sucrose | Ethanol |
| --- | --- | --- | --- |
| 1% | 3.968 | 4.416 | 3.062 |
| 2% | 5.296 | 5.402 | 1.548 |

**Table S2.** OD600 of Y-AH3/E3 after cultured in various carbon sources for 48 h

|  | Glucose | Sucrose | Ethanol |
| --- | --- | --- | --- |
| 1% | 4.282 | 4.422 | 2.204 |
| 2% | 5.678 | 5.712 | 2.15 |

**Table S3**. OD600 of Y-AH3/E3 after cultured in various carbon sources for 72 h

| Forward | Reverse | | | |
| --- | --- | --- | --- | --- |
|  | JH1 | JH2 | JH3 | JH4 |
| VH1 | 1 | 11 | 21 | 31 |
| VH2 | 2 | 12 | 22 | 32 |
| VH3 | 3 | 13 | 23 | 33 |
| VH4 | 4 | 14 | 24 | 34 |
| VH5 | 5 | 15 | 25 | 35 |
| VH6 | 6 | 16 | 26 | 36 |
| VH7 | 7 | 17 | 27 | 37 |
| VH8 | 8 | 18 | 28 | 38 |
| VH9 | 9 | 19 | 29 | 39 |
| VH10 | 10 | 20 | 30 | 40 |

**Table S4.** The corresponding numbers of VH PCR products generated by the indicated forward and reverse primers

| Forward | Reverse | | |
| --- | --- | --- | --- |
|  | JK1 | JK2 | JK3 |
| VK1 | 1 | 11 | 21 |
| VK2 | 2 | 12 | 22 |
| VK3 | 3 | 13 | 23 |
| VK4 | 4 | 14 | 24 |
| VK5 | 5 | 15 | 25 |
| VK6 | 6 | 16 | 26 |
| VK7 | 7 | 17 | 27 |
| VK8 | 8 | 18 | 28 |
| VK9 | 9 | 19 | 29 |
| VK10 | 10 | 20 | 30 |

**Table S5.** The corresponding numbers of VK PCR products generated by the indicated forward and reverse primers

**Table S6.** Gene alignment of the neutralizing clones isolated from the Fab library

| Clone | Gene usage | | | | | | |
| --- | --- | --- | --- | --- | --- | --- | --- |
|  | HV | HD | HJ | | KV | KJ | |
| Clone#3 | Homsap IGHV3-21*03 F | Homsap IGHD1-26*01 F | Homsap IGHJ1*01 F | Homsap IGKV4-1*01 F | | | Homsap IGKJ1*01 F, or  Homsap IGKJ3*01 F |
| Clone#7 | Homsap IGHV3-21*03 F | Homsap IGHD1-26*01 F | Homsap IGHJ1*01 F, or Homsap IGHJ2*01 F | Homsap IGKV2-29*03 F | | | Homsap IGKJ5*01 F |
